# Nutritional Influence on Epigenetic Marks and Effect on Livestock Production

**DOI:** 10.3389/fgene.2016.00182

**Published:** 2016-10-24

**Authors:** Brenda M. Murdoch, Gordon K. Murdoch, Sabrina Greenwood, Stephanie McKay

**Affiliations:** ^1^Department of Animal and Veterinary Science, University of Idaho, MoscowID, USA; ^2^Department of Animal and Veterinary Sciences, University of Vermont, BurlingtonVT, USA

**Keywords:** epigenetics, livestock, nutrients, nutritional epigenetics, methylation

## Abstract

Nutrition represents one of the greatest environmental determinants of an individual’s health. While nutrient quantity and quality impart direct effects, the interaction of nutrition with genetic and epigenetic modifications is often overlooked despite being shown to influence biological variation in mammals. Dissecting complex traits, such as those that are diet or nutrition related, to determine the genetic and epigenetic contributions toward a phenotype can be a formidable process. Epigenetic modifications add another layer of complexity as they do not change the DNA sequence itself but can affect transcription and are important mediators of gene expression and ensuing phenotypic variation. Altered carbohydrate metabolism and rates of fat and protein deposition resulting from diet-induced hypo- or hyper-methylation highlight the capability of nutritional epigenetics to influence livestock commodity quality and quantity. This interaction can yield either products tailored to consumer preference, such as marbling in meat cuts, or potentially increasing productivity and yield both in terms of carcass yield and/or offspring performance. Understanding how these and other desirable phenotypes result from epigenetic mechanisms will facilitate their inducible potential in livestock systems. Here, we discuss the establishment of the epigenome, examples of nutritional mediated alterations of epigenetics and epigenetic effects on livestock production.

## Introduction

In order to meet the dietary needs of the world’s projected population of 9.1 billion by 2050, the Food and Agriculture Organization estimates that food production must increase by 70% (FAO, 2009). Attaining this goal of increased food production, especially in developing countries, will require the adoption of more efficient and sustainable production methods. In addition, climate and environmental impacts will need to be considered in order to meet the estimated food production targets. Taking advantage of nutritional epigenetics to increase or improve livestock production can aid in sustaining and/or increasing food production. Nutritional epigenetics involves the study of epigenetic mechanisms related to gene-diet interactions (Park et al., 2012). Even though the effective utilization of nutritional epigenetics applied to livestock production is in its infancy, a foundation of nutritional epigenetics research has been established and has validated the direct relationship between nutrients, including vitamins, macronutrients, and phytochemicals, and epigenetic modifications mainly through regulation of methionine cycle substrate availability and enzyme activity. However, diet may not only play a regulatory role in the epigenetic makeup of the first generation consumer, it may also impact progeny performance. This could be a key driver for productivity improvement, particularly in systems such as the swine and poultry industry, where reproductive cycles are rapid with large offspring numbers. Initial steps in this research field have identified the impact of plane of nutrition of the dam on offspring phenotype. In an effort to elucidate the contribution of epigenetic inheritance toward complex traits we must also examine the potential of transgenerational epigenetic inheritance through establishment of the epigenome, as well as specific nutrient effects and nutritionally mediated impacts on epigenetics.

The pathways governing nutritional regulation of epigenetic modifications in turn can be considered at each level of the substrate input, the molecular target, as well as the endpoint product. Many molecular forms of epigenetic modification have been described including changes in chromatin packaging due to histone acetylation and methylation, repression of gene expression by non-coding RNA, and repression of gene transcription by DNA methylation. These epigenetic mechanisms, in addition to others, have been recently described in detail (Ibeagha-Awemu and Zhao, 2015; Triantaphyllopoulos et al., 2016). Epigenetic effects include chemical modifications to DNA base pairs that do not change the DNA sequence itself but can affect transcription, thus resulting in phenotypic variation. Ultimately, the primary goal is to understand how epigenetic mechanisms will allow for inducible desirable phenotypes. The purpose of this review is to highlight the interaction between nutrition and epigenetics with respect to traits of economic importance in livestock production.

## Establishment of the Epigenome

**FIGURE 1:**
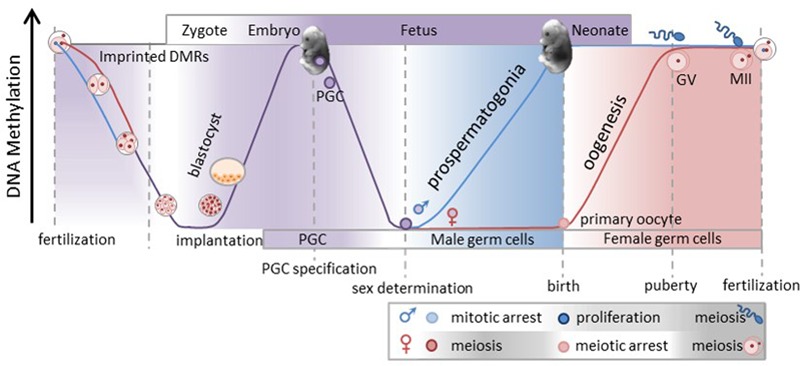
**The genome goes through two cycles or waves of epigenetic reprogramming.** At fertilization the paternal genome is actively demethylated (blue line), whereas the maternal genome is passively demethylated (red line). As a result most of the blastocyst genome is demethylated by implantation. However, some regions of the genome, like imprinted differentially methylated regions (DMRs) and intracisternal A particles (IAP’s), maintain methylation allowing some epigenetic information to be transmitted to the next generation (gray line). Subsequent to implantation the genome of the developing zygote goes through *de novo* methylation completing the first wave of reprogramming. The degree of purple shading indicates the degree of methylation in the genome. After the primordial germ cells (PGC) within the developing embryo are specified they will proliferate and migrate to the genital ridge and in doing so will go through genome demethylation. This marks the beginning of the second wave of reprogramming. Upon sex determination and gametogenesis the genome is remethylated at different times in each sex. Germ cells in the male gonad are remethylated in the developing embryo while *in utero* and the degree of methylation is denoted by blue shading. The prospermatogonia proliferate and at puberty continually progress through meiosis to eventually produce mature spermatozoa. Conversely, female germ cells entire meiosis unmethylated *in utero* and arrest in prophase 1 just before or at birth. During the follicular growth phase the oocyte genome will be remethylated, germinal vesicle will extrude the first polar body and if fertilized will complete MII. As a result of oocyte remethylation occurs during the follicular growth phase of only the specific oocytes when they are recruited. This figure was generated by combining and modifying images from the following related works: Data from Dean, W., Santos, F., and Reik, W., Seminars in Cell & Developmental Biology, 14, 93–100, Elsevier, 2002; Data from Cowley, M., and Oakey, R., Molecular Cell, 48, 819–821, 2012; Data from Smallwood, S. A. and Kelsey, G. Trends in Genetics, 28, 33–42, 2011; Data from Saadeh, H., and Schulz, R. Epigenetics and Chromatin, 7, 1–15, 2014.

To understand how epigenetic modifications mediate and affect gene expression it is important to understand how and when the epigenome is established. While we do not fully understand how all of the complex epigenetic genome changes occur, we do know that the genome is demethylated, or “erased,” and remethylated, yet some epigenetic information is retained and transmitted to the next generation. Furthermore, studies, primarily in mice, but also in cattle (Dean et al., 2003; Yang et al., 2007) have provided some understanding of the timing of these events. It is worth noting that when these important biological processes occur, i.e., when the epigenome of the progenitor cells are being erased and then re-established, represent critical times or “windows” in which nutritional changes or disruptions may be perpetuated. Studies have shown that perturbation, be they genetic or the result of nutritional changes, during these critical windows of time can directly affect the developing animal and/ or the cells that contribute to future generations. Here we will review and discuss the timing in which the genome undergoes the two different cycles or waves of epigenetic reprogramming (**Figure 1**).

At fertilization genetic material from two haploid gametes must come together to create a new diploid organism. Since the mid 1970’s scientists have understood that one germ cell from each sex are required for the development of a viable embryo. Pathological analyses of human germ cell tumors have provided important biological information regarding the difference of parental genetic origins. Through histopathological analyses of ovarian teratomas and hydatidiform mole, functional differences of what the maternal and paternal genome each contribute to cell growth and differentiation was recognized. Ovarian teratomas occur in the ovary and consist of three germinative layers (Linder et al., 1975) whereas hydatidiform mole occurs in the uterus and only contain extra trophoblast (Kajii and Ohama, 1977; Wake et al., 1978). However, both tumors arise from uniparental genomes. Genetic analyses have shown that the cells which result in ovarian teratomas consist only of the maternal genome whereas the hydatidiform mole is only the paternal genome. Furthermore, a series of pronuclear transfer studies in the early 1980’s using mice provided experimental evidence that the sex of the parentally derived genome directly affects the developing embryo (McGrath and Solter, 1984; Surani et al., 1984). These studies provided the early conceptual framework and evidence for two important concepts: first is that one of each parental germ cell is necessary and second is the understanding of what each of the maternal and parental genomes directly contribute toward specific cell linages the proper development of a viable fetus. While it may not be surprising that it is necessary to have both a maternal and paternal genome for the development of a viable embryo it does raise the question how are these genomes differentiated such that parent of origin is readily identified at fertilization.

At fertilization the parental genomes start to go through global demethylation where prior epigenetic programming is erased so that new totipotent cells can be reset for specific cell linage determination in a manner which results in the correct cellular differentiation. This is one cycle or wave of epigenetic reprogramming which notably occurs of the developing zygote within the maternal environment. However, because each of the parental genomes is necessary to contribute specifically to the different and specialized manner of the development of the zygote the demethylation changes occur in a manner specific to the parent of origin. The male pronucleus, for example, is rapidly and actively demethylated involving Ten–Eleven Translocation (TET) dioxygenases proteins (Tahiliani et al., 2009; Gu et al., 2011; Hackett et al., 2013), whereas the maternal genome is demethylated passively, i.e., methylation is lost via lack of DNA methylation maintenance during replication, resulting in a progressive loss of methylation at each cell division. After the formation of the zygote and by the blastocyst stage most, but not all, of the genome has undergone progressive demethylation and has been erased. However, some specific genomic regions escape post fertilization eraser or demethylation. For example, these regions include intracisternal A particles (IAPs) and imprinted genome differentially methylated regions (gDMRs). As a result, some parental allele specific methylation expression is transmitted to the next generation resulting in allele specific expression of associated imprinted genes.

After implantation the cell mass or blastocysts undergoes *de novo* methylation in a cell lineage specific fashion. DNA methylation increases in the cells which give rise to the embryo, a primitive ectoderm, but not the cells which give rise to the placenta and yolk sac, or primitive endoderm (Ficz et al., 2011). This process requires the cells to undergo both *de novo* methylation via DNA methyl transferase (DNMT), DNMT3a, DNMT3b, and DNMT3L (Okano et al., 1999; Suetake et al., 2004), as well as methylation maintenance via DNMT1. *Dnmt1* null mutant mice displayed embryo arrest and death, suggesting that it is essential that these epigenetic marks are properly maintained with continued cell division in a post implantation growing embryo. It is possible that both de- and re-methylation of specific genomic sites are necessary for both the control of transcriptional activity required for the developing zygote as well as the subsequent repression and therefore global silencing of retrotransposons. This essentially completes one cycle or wave of epigenetic reprogramming.

In the developing embryo it is important that a few cells undergo germ cell specification. These primordial germ cells (PGC) become the founder cell population which will ultimately give rise to the germ cell population. Signaling events by bone morphogenetic protein 4 (*BMP4*) induce the PGC to express B-lymphocyte-induced maturation protein-1 (*BLIPM1*) protein, and *BLIMP1* suppresses homeotic genes (HOX) which activate somatic cell differentiation (Saitou et al., 2002). These PGC require sex specific epigenetic reprogramming for the successful transition of gametogenesis. Germ cell specification marks another wave or cycle of methylation reprogramming. The proliferating PGC migrate to the genital ridge where they arrive, settle and become the gonad. Although at least 70% of somatic cells (Popp et al., 2010) and in particular, some genomic regions (IAPs and imprinted DMRs) are not demethylated, germ cells need to be reset so that they properly reflect the sex of the developing embryo. As a result the PGC are erased, overall methylation is generally less than 10% in PGCs (Popp et al., 2010) prior to sex determination so that the correct genes can subsequently be expressed to initiate proper sex specific development of gametogenesis. Once sex is determined and the proper cell lineages are set in the gonad, the genome is remethylated. However, when this remethylation happens is very different in male and female developing gonad. The differences between when the male and female epigenome is established is synonymous with the time frame wherein the cells that will give rise to the next generation are vulnerable to epigenetics changes and this also differs between the two sexes.

In the male embryo the germ cells will arrest in the first growth phase (G1) of mitosis and all the prospermatogonia will undergo remethylation before meiosis and birth. As such the maternal uterine environment is the direct influential environment of the developing male embryo and male primary germs cells remethylation. Subsequent to the establishment of the epigenome and birth, male germ cells will undergo multiple cell divisions, then meiosis prior to the development of mature sperm. Therefore, the methylation established in the early cell population needs to be maintained during cell replication post puberty and throughout the reproductive years. Finally, volume compacting necessary to produce the mature sperm is achieved by the replacement of approximately 85% of the histones with protamines in spermatozoa.

Conversely, in the female embryo, the PGCs remain in an unmethylated state. In the PGCs, and only the PGCs of the female embryo, there is a reduction in *Xist* RNA level to maintain two active X chromosomes for subsequent gamete formation. The female germ cells enter meiosis, complete pachytene and arrest at diplotene stage of anaphase 1 at or before birth unmethylated. It is not until after birth, during the follicular growth phase of the oocyte that the arrested primary oocytes are remethylated. Therefore, the critical window when the female genome is remethylated and sensitive to changes is during the follicular growth phase of those eggs. At puberty and in response to endocrine signals the germinal vesicle oocyte resumes meiosis and completes the first meiotic division. Subsequent to the extrusion of the first polar body and upon fertilization the second meiotic division (MII) will take place.

## Nutritional Mediated Alterations of Epigenetics

While mechanistically naïve to the epigenetic process, the oft considered “father of epigenetics”; Barker et al. (1989, 1990, 1993) reported anecdotal evidence of the heritable pattern linking maternal malnourishment with prevalence of heart disease, hypertension and type 2 diabetes in progeny. Many of these gene-environment interactions exert small over-all effects, however, others such as the hypomethylation and concomitant increase in expression of melanocortin 4 receptor are directly linked to obesity through stimulation of orectic signaling and thus energy intake (Farooqi and O’Rahilly, 2008). There is a dichotomy relating to lipid deposition between most mammals. Where excess adiposity is associated with disease in humans and biomedical model rodents; in livestock species selective and site specific adiposity is associated with valued productive traits such as reproduction, fecundity, fitness during times of reduced feed availability and/or cold as well as deposition of intramuscular fat as marbling.

It is well understood that there exists a substantial gene and environment interaction when considering phenotypic variation, and a key environmental player in these phenotypes is certainly nutrient quality and quantity. In fact, two significant examples of nutrient induced epigenetic events can be found in mice (Waterland and Jirtle, 2004) and honeybees (Kucharski et al., 2008). The agouti coat color phenotype in mice has been shown to be influenced by maternal diet (Waterland and Jirtle, 2004). Expression of the agouti gene in mice conveniently results in an observable yellow coat color and obesity. However, if mice have the A^vy^ locus and are fed a ration with sufficient methyl donating constituents; such as folate and betaine then the expression of the agouti gene is diminished and imprinting decreases both the yellow coat and the obesity. Agouti coat color in mice was the first experiment to show that maternal diet had the ability to affect epigenetic modifications and gene expression in offspring. Whereas, honeybees utilize differential nutrition with genetically identical larvae to generate their worker caste system (Kucharski et al., 2008). Both of the previously mentioned studies provide evidence that epigenetic events may be nutritionally induced.

Several reviews have outlined the various points of potential interaction between nutrition and epigenetics, including the impact of supplementation or biological deficiency of macronutrients, as well as specific micronutrients and secondary plant metabolites, on methylation (Choi and Friso, 2010; Anderson et al., 2012; Jiménez-Chillarón et al., 2012; Shankar et al., 2013). Logically, the main focus of nutritional epigenetics has been on the specific precursors, substrates and enzymes required in the folate cycle, the methionine cycle, and histone methylation, as these relationships are the most direct. Jiménez-Chillarón et al. (2012) outlined the impact of nutrients on methylation capacity by affecting (1) availability of nutrient-derived substrates for S-adenosylmethionine (SAM) synthesis, (2) pool size of nutrient-derived cofactors required for folate- and methionine- cycle completion, or (3) circulating concentrations of diet-derived regulators of DNMT expression and activity. Targeted dietary supplementation with folate, choline, or betaine appears to consistently increase DNA methylation because these nutrients are methyl group donors (Anderson et al., 2012; Crider et al., 2012). In addition to folate, supplementation with other B vitamins, notably B_2_, B_6_, and B_12_, also appears to increase DNA methylation because of their role as cofactors in the methylation process (Powers, 2003; Craig, 2004). Though choline and betaine are both involved in the conversion of homocysteine to methionine, supplementation with these downstream amino acids appears to produce a less consistent DNA methylation response (Waterland, 2006), but some success has been documented as outlined below.

Dietary induced epigenetic modifications have been studied in a variety of food animal or livestock species (**Table 1**). Nutrients such as folic acid and betaine have been shown to influence the function of enzymes that participate in the methylation process (Van den Veyver, 2002). Folic acid and betaine undergo a series of chemical reactions that result in the production of methionine (Anderson et al., 2012). Subsequently, methionine is converted to SAM, which donates methyl groups to DNMTs. The DNMTs then covalently attach the methyl groups to the five position of a cytosine base. This suggests that DNA methylation efficiency could be affected by animal nutrition as factors involved in the biochemical pathway that converts nutrients into SAM. When methyl donors such as folate or betaine are restricted from diet the observation of hypomethylation is generally anticipated. This result was observed in a study performed in Scottish blackface ewes where the extent of periconceptional availability of nutrients linked to the methionine-folate cycle altered DNA methylation (Sinclair et al., 2007). The nutrient deficient diet included restriction of vitamin B_12_, folate and methionine. At 12 months of age there was no measureable difference between offspring of ewes fed the control diet and the methyl-deficient diet. However, at 22 months, male progeny of the ewes fed the methyl-deficient diet had an estimated 25% greater body fat compared to the control diet offspring. As expected, dietary induced epigenetic modifications were found, and a majority of those were unmethylated or hypomethylated CpGs associated with the methyl deficient animals. Of perhaps more interest is that 53% of the altered loci were specific to male offspring whereas only 12% of the altered loci were specific to female offspring.

**Table 1:** Examples of nutritional mediated alterations of epigenetics in livestock.

Dietary change	Epigenetic effects	Outcome	Species	Reference
Vitamin B_12_, folate and methionine deficient	DNA methylation	Hypomethylation in methyl-deficient diet	Sheep	Sinclair et al., 2007
Betaine supplementation	DNA methylation	Increased global measures of DNA methylation	Chicks	Hu et al., 2015
Betaine supplementation	DNA methylation, Histone modification	*PEPCK1* hypomethylated and enriched for H3K27me3. *PEPCK2* and *FBP1* hypermethylated and enriched for H3K4me3	Neonatal Piglets	Cai et al., 2014
Zinc supplementation	DNA methylation	Hypomethylation and increased expression of gene *A20*	Hens	Li et al., 2015
Variable zinc levels	DNA methylation	Hypermethylation in regulatory regions of gene *ZIP4*	Piglets	Karweina et al., 2015
Restrictive feeding	Histone modification	Increased global measures of H3K9 acetylation and reduction in H3K9me3 in IUGR pigs subjected to restrictive feeding	Pigs	Nebendahl et al., 2016
Folic acid supplementation	DNA methylation	Hypermethylation in IUGR piglets with folic acid supplementation compared to piglets fed control diet	Pigs	Jing-bo et al., 2013
Maternal protein insufficiency	DNA methylation	Hypomethylation in *POMC* and *GR* in the sheep brain	Sheep	Begum et al., 2012
Methylating micronutrients	DNA methylation	*IYD* gene differentially methylated between groups of pigs with extreme obesity related phenotypes	Pigs	Braunschweig et al., 2012

Betaine has been utilized in livestock and chickens as a feed additive to improve a variety of economically important traits (Eklund et al., 2005; Alirezaei et al., 2012). Furthermore, dietary supplementation of methyl donors such as betaine generally results in global DNA hypermethylation. Although, recent publications pertaining to dietary induced epigenetic modifications from supplemental betaine have shown both hyper and hypomethylation in specific genes (Cai et al., 2014; Hu et al., 2015). An investigation of cholesterol metabolism through betaine supplementation in newly hatched chicks showed increased protein content levels of DNMT1 and thus increased measures of global genomic DNA methylation (Hu et al., 2015). However, when specific gene promoters were investigated for CpG methylation levels contrasting results were found. Increased methylation was found in the promoter regions of cholesterol-7alpha-hydroxylase, (*CYP7A1)* and sterol regulatory element binding protein 1*(SREBP1)* while decreased methylation levels were found in the promoter region of ATP binding cassette sub-family A member 1 (*ABCA1)*. In addition to studying the effects of betaine supplementation on methylation, Hu et al. (2015) investigated the relationship between betaine supplementation and histone methylation. Histone mark, H3 lysine 27 trimethylation (H3K27me3) generally tends to be associated with inactive gene promoters and therefore would be expected to be upregulated in the presence of hypermethylation (Davison et al., 2009). However, Hu et al. (2015) was unable to examine expression of H3K27me3 and instead looked at protein content levels. A decreased protein level of H3K27me3 was found in the liver of betaine-treated chicks. Ultimately, these results are not what one would expect and the authors propose a variety of environmental effects as the potential source of this inconsistency. In an another study, genes associated with key enzymes that regulate gluconeogenesis were evaluated for methylation, expression and histone status in piglets whose sows were fed betaine supplemented diets throughout pregnancy (Cai et al., 2014). Phosphoenolpyruvate carboxykinase 1 (*PEPCK1)* gene promoter was found to be hypermethylated but promoter regions of phosphoenolpyruvate carboxykinase 2 (*PEPCK2)* and fructose-1, 6-bisphosphatase (*FBP1)* were hypomethylated in livers of piglets prenatally exposed to betaine. As expected the methylation levels were inversely proportional with expression levels. When examining histone enrichment, *PEPCK1* was found to be enriched for H3K27me3 while *PEPCK2* and *FBP1* were found to be enriched for histone mark H3 lysine 4 trimethylation (H3K4me3). These results are not surprising, given that histone mark H3K4me3 tends to be associated with gene activation. Cai et al. (2014) have shown that the interaction of DNA methylation and histone modifications result in modified gene expression.

The effects of dietary supplementation of certain microelements relative to epigenetic modifications, such as zinc (Zn), have only recently been studied (Sharif et al., 2012). Current thinking of Zn relative to the methylation process is that Zn deficiency may limit the production of betaine-homocysteine-*S*-methyltransferase (BMHT). BMHT catalyzes the transfer of methyl groups from betaine to homocysteine, a key component of one-carbon metabolism (Anderson et al., 2012), and is a precursor to SAM. It is possible that Zn deficient diets may hinder the production of SAM which in turn would limit SAM’s methyl donor function. A limited number of studies have been published that investigate the dietary effects of Zn relative to DNA methylation and expression. Epigenetic effects in the promoter region of gene *A20*, an anti-inflammatory protein, was investigated in progeny of hens whose dietary Zn intake had been altered (Li et al., 2015). The authors show evidence that increased levels of Zn in the maternal diet lead to hypomethylation and subsequently increased expression of gene *A20* in offspring. In another gene specific study involving DNA methylation and Zn, alternative results were found. A study involving three groups of piglets, each with a different dietary Zn level, found that DNA methylation levels increased in regulatory regions of the gene *ZIP4* (Karweina et al., 2015), a zinc transporter involved in the uptake of Zn in the small intestine. The authors go on to confirm a negative correlation between dietary Zn concentrations and expression levels of *ZIP4*. Dietary Zn levels have shown varying response on methylation in each of these gene specific studies. Until a methylation based genome-wide analysis is performed, we will not be able to draw tangible conclusions regarding the effects of Zn on DNA methylation.

Another nutritionally mediated impact on epigenetics is that induced through maternal dietary protein insufficiency. This research overlaps with studies evaluating imprinting associated with “intrauterine growth retardation (IUGR)” since maternal protein insufficiency generally manifests as reduced birth weight of offspring. Low birthweight associated with protein insufficiency is correlated with elevated risk for hypertension, hepatic steatosis, cardiovascular disease and leptin and insulin resistance (Mazzio and Soliman, 2014). When methylation was examined on a candidate gene basis between IUGR and normal birthweight pigs, increased methylation was observed in the promoter region of two genes, fibroblast growth factor receptor 4 (*FGFR4*) and protein tyrosine phosphatase, receptor type, S (*PTPRS*) (Nebendahl et al., 2013). Subsequently, histone modifications were examined in tissues derived from the same group of pigs (Nebendahl et al., 2016). Key findings include increased global measures of (histone mark H3 lysine 9) H3K9 acetylation and reduction in H3K9me3 in IUGR pigs that underwent feed restriction. These results imply that feed restrictions or refeeding could alter histone modifications and potentially birthweight. In fact, folic acid supplementation in the diets of post weaning IUGR piglets has been shown to alter methylation in promoter regions of peroxisome proliferator-activated receptor alpha (*PPAR*α) and glucocorticoid receptor (GR). IUGR piglets who were fed a control diet showed evidence of hypomethylation but IUGR piglets whose diet was supplemented with folic acid showed the same methylation levels as normal birthweight piglets (Jing-bo et al., 2013). More targeted approaches of maternal protein insufficiency reveal that is *liver X receptor*α is hypermethylated (van Straten et al., 2010), as is hepatocyte nuclear factor 4 alpha (*HNF4a*) in pancreatic cells (Sandovici et al., 2011). Though, hypomethylation is reported in leptin in adipose (Jousse et al., 2011) and proopiomelanocortin (*POMC*) and GR in the sheep brain (Begum et al., 2012). The essential nature of food, especially in developing offspring should prepare us for the fact that we have likely only scratched the surface of genes that are imprinted in the face of varied nutritional status and quality. This is even truer in regards to the evaluation and understanding of these events and their effects in livestock.

An interesting period for study is the association between methylation at birth and frequency of developing later adiposity. Godfrey et al. (2011) report that methylation of the retinoid X receptor alpha (*RXR-alpha*) gene explains as much as 26% of childhood obesity. Furthermore, there is limited evidence that suggests that the maternal dietary lipid profile impacts *RXR-alpha* gene and *PPAR* gene methylation (Waterland and Rached, 2006). This anecdotally implies that the maternal dietary lipid composition may imprint genes that influence the probability of adolescent obesity. Obesity related traits have been studied in pigs where boars in the founding generation were either fed a control diet or a diet supplemented with methylating micronutrients (Braunschweig et al., 2012). Differentially expressed genes associated with back fat percentage, adipose tissue and fat thickness at the 10th rib, were detected in muscle and liver of F2 generation pigs and pathway analysis indicated that lipid metabolism and metabolic pathways were overrepresented. Of the six differentially expressed genes that were investigated for differential methylation, only the iodotyrosine deiodinase, *IYD*, gene was found to be significantly differentially methylated between groups of pigs. By far, the most ambitious obesity study is a recent summary of 46 genome-wide association studies of epigenetics in humans which has made some interesting discoveries. For instance, in progeny of child bearing obese women before and after bariatric surgery to dramatically reduce intake, the methylation of the promoter of the *PGC1-*α gene decreased after surgery and increased after surgery in the *PDK4* (van Dijk et al., 2015). Given that PGC1-α is a key regulator of energy metabolism and mitochondrial biogenesis, the decreased methylation of its promoter may reduce the propensity of metabolism in these offspring due to their higher metabolism. Pyruvate dehydrogenase lipoamide kinase isozyme 4, the gene product of *PDK4*, is a mitochondrial enzyme that regulates glucose and lipid homeostasis and therefore with hypermethylation and concomitant decreased expression, it is less clear how this may impact the energy balance and obesity of these offspring. Until genome wide methylation based approaches are undertaken in livestock species, our understanding of methylation as a mechanistic link between genotype and phenotype will remain insufficient.

Many studies have examined the variation in propensity for obesity associated with intake of a high-fat diet (HFD). A comparison of isocaloric high-fat and low fat rations demonstrated that the expression of several genes associated with lipid synthesis are altered in the HFD treated group, namely there is an increase in methylation in the promoter domains of fatty acid synthase (*FASN)*, and a mitochondrial protein of the electron transport chain (*NDUFB6*) (Lomba et al., 2009). In the brains of rodent species, maternal HFD consistently triggers durable epigenetic changes in genes that control regulation of intake, such as; opioid receptors (Plagemann et al., 2010; Vucetic et al., 2010a, 2011) and dopaminergic pathways (Vucetic et al., 2010b). Peripheral tissues of metabolic importance are also imprinted in rodent species by maternal HFD treatment, for instance pancreatic β-cell interleukin 13 receptor, alpha 3 (*Il13ra3)* and hepatic cyclin-dependent kinase inhibitor 1A (*cdkn1a)* both are decreased in methylation and are associated with elevated risks for metabolic syndrome (Jiménez-Chillarón et al., 2012). There is very limited evaluation of the effects of high caloric and/or HFD rations in livestock species and this is especially true in ruminants. This represents both a challenge and opportunity for future research targeting optimal maternal nutrition for desired progeny phenotypes.

Of practical importance for livestock production systems would be an improved understanding of the relationship between nutrient imprinting and growth attributes of progeny. A good example of this is the methylation of insulin-like growth factor 2 (*IGF2*) that was initially linked to the Dutch famine (Barker et al., 1993; Heijmans et al., 2008), and later in pigs and cattle (Van Laere et al., 2003; Berkowicz et al., 2012). Van Laere et al. (2003) demonstrated that a nucleotide substitution in an evolutionarily conserved CpG Island within intron 3 of the *IGF2* gene was the causal mutation underlying a QTL for muscle growth in the pig. This point mutation resulted in a gel mobility shift within subsequent electrophoretic mobility shift assays (EMSA) experiments, suggesting that a mutation to this region recruits different transcription protein complexes (Van Laere et al., 2003). Given that the product of this gene plays a significant physiological role in reproduction, milk production and growth it stands to reason that it is important to consider this in any production system. Liu et al. (2011) examined how dietary protein in the maternal ration of Meishan pigs influenced the myostatin gene. They determined that the myostatin gene expression in low protein offspring was down-regulated at weaning but up-regulated at finishing phase. What came to light is that the histone acetylation of the myostatin gene promoter varied. Low protein piglets showed decreased binding of the CCAAT/enhancer-binding protein β (C/EBPβ) at three target locations in the myostatin promoter. In contrast, at the finishing phase, histone H3 acetylation and histone H3 lysine 27 trimethylation were increased in low protein progeny pigs increasing C/EBPβ binding (Liu et al., 2011). This is not the whole story though as skeletal muscle growth is also affected by several microRNA and maternal low protein diets resulted in decreased expression of *Sus scrofa* microRNAs 136 and 500 (ssc-miR-136 and ssc-miR-500) (Liu et al., 2011). Therefore, to no surprise, a polygenic trait such as muscle growth needs to be evaluated temporally but also across all levels of imprinting and expression regulation, as well as indirect and epistatic events. What can be concluded is that nutrition impacts epigenetic imprinting, and imprinting affects important production traits such as growth, thus improved research and understanding is both useful and warranted.

## Epigenetic Effect on Livestock Production

In livestock genomics, nutritional phenotypes have been studied primarily from the perspectives of growth, feed intake, and efficiency. Associations between epigenetic modifications and nutritional phenotypes have been characterized in livestock in individual genes (Van Laere et al., 2003) and in genome-wide nutritional epigenomic studies (Li et al., 2012; Shin et al., 2014). While it has been discovered that DNA methylation is associated with phenotypic variation of nutritionally related phenotypes (Li et al., 2012; McKay, 2015), the extent of phenotypic variation that can be accounted for by epigenetic modification remains unknown. Associations between genetic variation, epigenetic mechanisms and subsequently economically important phenotypes have previously been identified in livestock. The *callipyge* phenotype in sheep is only expressed in heterozygotes that inherit the mutation from their paternal parent. Vuocolo et al. (2007) found atypical expression of several imprinted genes in a 1 Mb region containing the *callipyge* mutation and implicated histone modifications as the epigenetic factor responsible for the phenotype (Vuocolo et al., 2007). In addition, it has also been shown that DNA variants, frequently in the form of single nucleotide polymorphisms (SNPs), influence the level of methylation (Gibbs et al., 2010). When Gibbs et al. (2010) examined the extent of genetic control of DNA methylation, a large number of *cis-*methylation qualitative trait loci (*cis*-meQTL) were reported. In this context, a *cis-*meQTL is defined as a genomic region that includes SNPs within 1 Mb of the CpG site associated with methylation state. It has been demonstrated that SNP variation strongly influences the level of DNA methylation at sites that contain intermediate levels of methylation. These studies demonstrate that elements of DNA sequence can affect methylation levels which in turn affect phenotypic variation. Now, the question exists as to how the livestock community can utilize epigenetic alterations for production of economically important phenotypes. While this subject has been discussed, there seems to be disparity regarding the matter (Goddard and Whitelaw, 2014). Investigating environmental effects on DNA methylation is essential to resolving the unknown extent of epigenetic effects on phenotypic variation of economically important traits.

One of the overarching goals of the livestock genomics community is investigating economically important traits to determine the extent to which phenotypic variation can be accounted for by genetic variation. Technological advances in recent years have facilitated the dissection of economically important traits in order to uncover the underlying mechanisms that are driving phenotypic variation. However, in order to completely comprehend epigenetic modifications as a mechanistic link between genotype and phenotype, we must generate tools that allow us to interrogate livestock epigenomes in a more cost effective manner. Epigenetic studies in livestock animals have utilized DNA methylation based next generation sequencing methodologies in order to explore the contribution of methylation toward the phenotypic variation of their respective economically important traits (Li et al., 2012; Doherty and Couldrey, 2014; McKay, 2015; Boddicker et al., 2016). Although the cost of genomic sequencing has declined since 2001 (Wetterstrand, 2015), the cost of methylation based next generation sequencing methods has not. Subsequently, our ability to perform epigenome-wide association studies (EWAS) is greatly inhibited. Epigenome-wide association studies are a vital analysis necessary for determining the epigenetic contribution toward phenotypic variation in economically important traits and diseases. Utilizing high resolution DNA methylation arrays, as has been done in humans (Bibikova et al., 2011), will enhance the ability of the livestock industry to perform EWAS and permit the resolution of mechanisms underlying epigenetic inheritance and possibly enable the livestock industry to utilize epigenetic modifications for livestock production and breeding (Rolf et al., 2014).

## Conclusion

The ability of nutrients to alter epigenetic modifications and subsequently phenotypic variation has been well documented. Ultimately, the livestock community would like to manipulate environmental factors, such as nutrients, to consequently yield the epigenetic mechanisms necessary to achieve phenotypes of interest. Diet supplementation or restriction of choline, betaine, folate, B2, B6, B12, and zinc have all been met with some level of success in inducing epigenetic modifications, likely by their respective roles in the folate cycle, methionine cycle, or histone methylation process. Improved or increased livestock productivity using nutritional epigenetics has been observed through lower body fat (sheep) and higher protein mass (chicken) gains as well as altered expression of key gluconeogenic enzymes (pigs). In mammalian species, additional research to discern the role of environmental factors such as nutrients and genomic sequence effects on epigenetic mechanism is essential, especially at critical developmental time points. Generation of new tools and methodologies for interrogating the livestock epigenome will assist in disentangling genetic and epigenetic effects of complex traits. Ultimately, utilizing nutritional epigenetics for increasing and/or sustaining food production will require additional research.

## Author Contributions

All authors listed, have made substantial, direct and intellectual contribution to the work, and approved it for publication.

## Conflict of Interest Statement

The authors declare that the research was conducted in the absence of any commercial or financial relationships that could be construed as a potential conflict of interest.

The reviewer SK and handling Editor declared their shared affiliation, and the handling Editor states that the process nevertheless met the standards of a fair and objective review.
